# Unsupervised Method Based on Superpixel Segmentation for Corpus Callosum Parcellation in MRI Scans

**DOI:** 10.1007/978-3-030-51517-1_10

**Published:** 2020-05-31

**Authors:** Amal Jlassi, Khaoula ElBedoui, Walid Barhoumi, Chokri Maktouf

**Affiliations:** 8 Digital Research Centre of Sfax, Sfax, Tunisia; 9 Institut Mines-Télécom, CNRS, Paris, France; 10 Université de Sherbrooke, Sherbrooke, QC Canada; 11 Digital Research Centre of Sfax, Sfax, Tunisia; 12 University of Sfax, Sfax, Tunisia; 13 Institut Supérieur d’Informatique, Research Team on Intelligent Systems in Imaging and Artificial Vision (SIIVA), LR16ES06 Laboratoire de recherche en Informatique, Modélisation et Traitement de l’Information et de la Connaissance (LIMTIC), Université de Tunis El Manar, Tunis, Tunisia; 14 Université de Carthage, Ecole Nationale d’Ingénieurs de Carthage, Tunis, Tunisia; 15 Nuclear Medicine Department, Pasteur Institute of Tunis, Tunis, Tunisia

**Keywords:** Corpus callosum, MRI, Parcellation, Superpixel

## Abstract

In this paper, we introduce an unsupervised method for the parcellation of the Corpus Callosum (CC) from MRI images. Since there are no visible landmarks within the structure that explicit its parcels, non-geometric CC parcellation is a challenging task especially that almost of proposed methods are geometric or data-based. In fact, in order to subdivide the CC from brain sagittal MRI scans, we adopt the probabilistic neural network as a clustering technique. Then, we use a cluster validity measure based on the maximum entropy (Vmep) to obtain the optimal number of classes. After that, we obtain the isolated CC that we parcel automatically using SLIC (Simple Linear Iterative Clustering) as superpixel segmentation technique. The obtained results on two challenging public datasets prove the performance of the proposed method against geometric methods from the state of the art. Indeed, as best as we know, it is the first work that investigates the validation of a CC parcellation method on ground-truth datasets using many objective metrics.

## Introduction

**Fig. 1.**
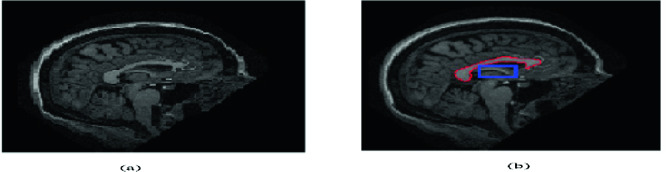
Example of sagittal brain MRI slices from the OASIS dataset: (a) The input MRI. (b) Delineation of the CC, where the fornix (framed in blue) appears in the neighborhood of the CC while being of similar appearance. (Color figure online)

Thanks to advances in magnetic resonance imaging, neuroscientists and clinicians can study in depth the Corpus Callosum (CC) and mainly the correlation between the CC’s dimensions and some neurological diseases. The CC, which is the largest white matter structure and the biggest fiber tract connecting corresponding regions of the cerebral cortex in the two cerebral hemispheres, integrates motor, sensory, and cognitive functions of the brain [1]. Anatomically, more than half of the axons composing the CC are surrounded by myelin, which gives this structure its remarkable appearance in midsagittal T1-weighted MRI images. However, in many sagittal brain MRI slices, the fornix appears in the neighborhood of the CC with a similar intensity (Fig. 1) [2]. The CC is usually divided into smaller regions such as rostrum, genu, body, and splenium. This subdivision of the CC is called parcellation and it is proving to be very useful for an effective analysis of the CC [2, 3]. In fact, the CC shape may be the cause of many neurodegenerative diseases such as epilepsy, alzheimer, autism, depression and other types of psychosis [4]. The CC analysis is also important for studying aging, gender differences and laterality [5]. Hence, various studies have evaluated shape or volume variation of the CC parcels. They revealed a correlation between CC’s abnormalities and many diseases. For instance, [6] shows that the rate of change in CC or one of its sub-regions is more closely associated with the progression of Alzheimer’s disease. Moreover, the CC parceling can be an appropriate group biomarker for an objective evaluation of treatments aimed at slowing the progression of Alzheimer [7]. Furthermore, several works have identified volume alterations of the CC and its sub-regions in subjects with Autism Spectrum Disorders (ASD). In this context, a study of the CC volume of 40 pre-schoolers, with different sex and age, suffering from ASD was made by applying the “FreeSurfer” automated parcellation software. This study demonstrated that the total volume of the CC and its sub-regions is correlated with autism severity [8]. Another study conducted on 75 participants with Parkinson Disease (PD) and 24 Healthy Control (HC) confirms that CC sub-regions abnormalities might be the cause of Parkinson disease. Indeed, participants with PD showed an increase in the 3 anterior callosal segments compared to HC [9].

Generally, the CC parcellation into callosal regions allows for a precise differentiation of motor connectivity and the structural integrity of these tracts in the CC [10]. Thus, the CC parcellation should be so helpful to better understand inter-hemispherical callosal connectivity in patients or healthy subjects [11]. In particular, MRI takes advantage of the macroscopic geometrical arrangement of white matter bundles that it makes capable of generating good CC visualization from the sagittal plane. In any way, the parcellation of the CC stills an important task for radiologic assessment despite there are no real or visible borders to allow this subdivision. Nevertheless, the visual inspection of CC structures in MRI scans suffers from both inter- and intra-specialist variability. On the one hand, the manual CC segmentation methods require strongly visual effort, specialized training skill, and are time-consuming processes. On the other hand, several geometrical methods for the CC parcellation have been proposed such as Witelson and Hofer methods [12]. However, these methods cannot be satisfactorily validated due to the lack of qualitative parameters and reference standards. Although all these difficulties, the development of an automatic CC parcellation method is an inescapable need to ensure a reliable diagnosis. Such parcellation is so independent from the operator skills and may be extended to other brain structures parcellation. Thus, since there are no visible landmarks indicating where the CC should be subdivided, the development of a fully automatic CC parcellation method is highly challenging, even for specialists. To deal with this issue, we propose to automatically parcel the CC within MRI images. By validating it, for the first time, on large and public datasets, the proposed method records promising results. In fact, the contribution of this work is twofold:

- As best as we know, we adopt for the first time the superpixel segmentation algorithm called Simple Linear Iterative Clustering (SLIC) for the CC parcellation [13]. Despite its simplicity, SLIC has been demonstrated to be effective in various computer vision applications [14].
- The subdivision process of the proposed method is fully automatic and it is the second study that proposed a non-geometric analysis for the CC parcels, to the best of our knowledge [15]. Although it is based only on the MRI data of each analyzed subject, with no parameter adjusting, the proposed method proved quantitatively its superiority over state-of-the-art methods.

The rest of this paper is organized as follows. In Sect. 2, we briefly review existing methods for the CC parcellation. Section 3 presents the proposed method based on SLIC. Experimental results are discussed in Sect. 4. The last section concludes the paper and points some directions for future work.

## Related Work

**Fig. 2.**
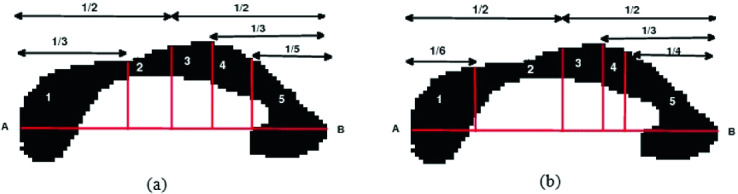
CC geometric parcellation with divisions presenting the five regions (on an MRI scan from the OASIS dataset) using the method of: (a) Witelson. (b) Hofer.

Few CC parcellation methods were proposed. However, most of these methods have not surmounted all the challenges encountered. In fact, the CC parcellation is a challenging task given that a normal shape of the CC might not clearly highlight all parcels, what can increase the diagnosis complexity. In addition, many internal abnormalities might include bumps which are hard to detect. Existing CC parcellation methods can be divided into two main classes: geometric methods and non-geometric ones. On the one hand, since there are no real or visible boundaries allowing the CC parcellation, several geometrical methods were presented to perform this task. Among these methods, two particular ones are widely adopted. The first was proposed by Witelson and it is based on postmortem connectivity analysis in primates and humans [16]. This method divides the CC into five regions ranging from anterior dimension to the posterior dimension. The CC subdivision is done into an anterior third, the middle of the anterior and posterior midbody, a posterior third and the posterior one-fifth. The rostrum, genu, and rostral body presenting the regions of the anterior third illustrate the prefrontal, premotor, and supplementary motor cortical areas. However, the posterior midbody is crossed by the somaesthesic and posterior parietal fiber bundles. The sub-regions of the posterior third, containing the isthmus and splenium, are allocated to temporal, parietal, and occipital cortical regions. Thus, this parcellation method, and as any geometric methods, neither reflects the real texture nor the internal organization of the CC. In addition, the CC parcellation is strongly dependent on the brain conservation process, since it is based on post-mortem data. Differently, Hofer proposed the only work based on tractography of DTI (Diffusion Tensor Imaging) by subdividing the CC into five regions from an average behavior observed via tractography in a specific population of 8 subjects [1]. As already proposed by Witelson, the geometric baseline in the midsagittal section of the CC is defining the anterior and posterior points of the structure. The first region, which represents the first sixth, contains fibers projected in the prefrontal region. The remainder of the anterior half CC illustrates the second region containing the fibers that form the motor and motor areas of the cerebral cortex. In fact, these fibers form together the largest CC region and are placed in the back section of the structure. The third region presents the posterior half minus the posterior third. It contains fibers responsible for the primary motor cortex. However, this part of the parcellation scheme is in conflict with Witelson’s method. The fourth region forms third minus the posterior quarter, presenting the primary sensory fibers. The last and the fifth region represents the CC posterior quarter crossed by the parietal, temporal and visual fibers. Figure 2 shows a comparison between the geometric schemes proposed by Witelson and Hofer. We notice that geometric methods allow only to divide the CC into the same regions among all subjects without considering the human and individual brain features between different subjects. On the second hand, differently to geometric parcellation methods, Rittner proposed a data-driven method based on the Watershed technique [15]. This method is composed of four steps. The first step consists in the weighting of the fractional anisotropy. The second step performs the selection of the brain midsagittal plane, followed by the third and the last step which are the CC segmentation using the Watershed technique, and its parcellation with fixed markers. Nevertheless, this method suffers from sensitivity to parameters selection. In order to overcome its limitations, Cover extended the Rittner method with some important changes [12]. Practically, the author replaced all steps except the first step in order to lead to a more robust data-driven method. Indeed, the parcellation is improved by applying the K-means algorithm after defining the CC centerline. When comparing this method to that of Rittner, and although both are based on Watershed, it is confirmed that this method had a better generalization ability using no fixed markers to execute the Watershed transform. However, due to the lack of quantitative metrics and reference standards, these methods cannot be correctly validated.

## Proposed Method

Differently to existing methods, we propose a subdivision scheme that considers only the MRI data [14]. Using the SLIC superpixel segmentation technique, the method is composed of two main steps: CC segmentation and CC parcellation. This comes from that the SLIC presents one of the most popular images over segmentations that is commonly used as supporting regions for primitives to reduce computations in various computer vision tasks.

### CC Segmentation of the Midsagittal Slice

**Fig. 3.**
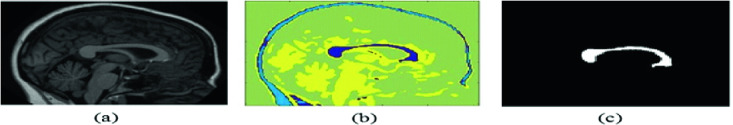
CC segmentation: (a) Input sagittal MRI. (b) Cluster Map. (c) Isolated CC.

We adopt herein our previous method [17] for the automatic CC segmentation of MRI sagittal section. It includes three main steps: image preprocessing using the Anisotropic Diffusion Filtering (ADF), classification based on the unsupervised Probabilistic Neural Network (PNN) classifier, and CC isolation using a spatial filtering (Fig. 3). In fact, the first step aims to enhance the signal-to-noise ratio by eliminating unwanted parts in the background and smoothing the internal part of the region while preserving its borders. In fact, ADF allows to unblock high-frequency noise while preserving the main edges of structures [18]. Then, the classification step permits to define the target classes using K-means, before classifying them by the PNN [17]. Thereafter, the Vmep index, which is based on the maximum entropy principle as an evaluation method that is called the cluster validity, is applied in order to determine the optimal number of clusters. The optimal number of classes is obtained when the Vmep validity index reaches its maximum value. This number is adopted for the PNN classification process to obtain the final cluster map. Once the CC class is identified, the CC region will be isolated by a spatial-based filtering. Finally, we defined the CC contour by applying a follow-up algorithm on the border pixels of the CC region that are characterized by a maximum of the spatial gradient [19].

### CC Parcellation

We propose a CC parcellation method based on SLIC, which is non-geometric and fully automatic superpixel segmentation technique. It works with no parameter adjusting and with no instantaneous training, leading to a more robust technique. Thus, in order to segment the CC into a set of superpixels, which refer to groups of pixels that represent perceptually significant small defined regions, we adopt the SLIC technique. It is an arrangement of K-means for superpixel generation in order to be faster than existing methods, more memory efficient while improving significantly the segmentation accuracy. It allows two important directions [14]. Firstly, it reduces greatly the number of distance calculations by restricting the search space to a region corresponding to the superpixel size. Therefore, a reduction in the complexity of being linear is achieved in the pixels’ number *N* and superpixels’ number *K* that is independent and user-defined. In our case, *N* and *K* are equal to 256 and 200, respectively. Secondly, a combination of color and spatial proximity is reached by a weighted distance measure that allows both controls over the size and compactness of the superpixels. Thus, each slice of the input MRI image is partitioned into different size regions. In fact, the initial grid size is defined as *S* (1). From the geometric center, the center superpixel of each region is computed. This geometrical center of each region is recursively updated in each iteration.

1 $$\begin{aligned} S = \sqrt{ \frac{N}{K}}. \end{aligned}$$

**Fig. 4.**
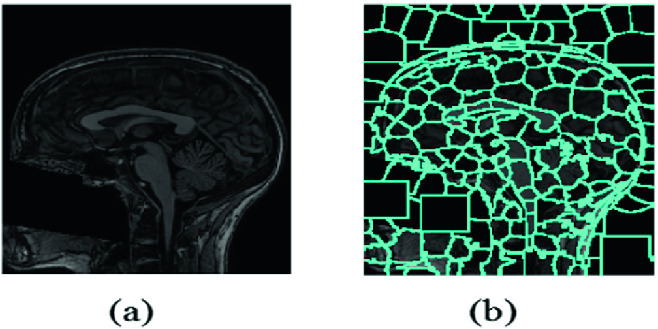
SLIC-based parcellation: (a) The input MRI. (b) Result of the SLIC method.

In order to regroup the pixel, both spatial and intensity distances are used. The spatial distance between the pixels *i* and *j* is defined as follows (2):

2 $$\begin{aligned} S_d = \sqrt{\left( {p_j - p_i } \right) ^2 + \left( {q_j - q_i } \right) ^2 }, \end{aligned}$$

where the coordinate values of pixel *i* and *j* are represented by *p* and *q*. The Eq. 3 calculates the intensity distance.

3 $$\begin{aligned} I_d = \sqrt{N_j + N_i }, \end{aligned}$$

where $N_j$ and $N_i$ represent the normalized intensity of pixel *j* and *i*, respectively. Equation 4 defines the combined distance measure $C_d$ of spatial and intensity.

4 $$\begin{aligned} C_d = \sqrt{I_d^2 .\left( {\frac{{S_d }}{S}} \right) ^2 + e^2 }, \end{aligned}$$

where *e* denotes the compactness coefficient. In fact, larger value of *e* illustrates more compact segments, whereas lower value of *e* represents flexible boundaries. The compactness coefficient is fixed in the range of $[0,\ 1]$. The superpixel computation of the proposed method is shown in Fig. 4.

## Experimental Results

For the evaluation of the proposed parcellation method, we are the only study that used brain MRI scans from two public datasets. On the one hand, we used the Open Access Series of Imaging Studies (OASIS) dataset, which is freely available on www.oasis-brains.org. It is created by Washington University Alzheimer’s disease Research Centre. This MRI dataset included a longitudinal collection of 416 subjects aged between 18 and 96 years, men and women, including 100 individuals with very mild to moderate Alzheimer’s disease (AD). All images were acquired on the same scanner using the same sequences. Each subject was scanned on two or more visits, separated by at least one year for a total of 373 imaging sessions. Each MR image within this dataset is composed of 128 slices with a resolution of $256 \times 256$ ($1\times 1mm$). In this work, we selected 1806 sagittal images that are qualified by a quality control according to severe artifacts. On the other hand, Autism Brain Imaging Data Exchange (ABIDE) is also investigated. In order to accelerate understanding of the neural bases of autism, the ABIDE dataset has supplied functional and structural brain imaging data collected from laboratories around the world. This dataset is composed of two large-scale collections called ABIDE-I and ABIDE-II. Each collection was collected independently across more than 24 international brain imaging laboratories. Thus, we generate a total of 2200 sagittal images with a resolution of $256 \times 256$. It is worthy noting that we have a challenging heterogeneous set of images of normal subjects and individuals with Autism and Alzheimer.

### Qualitative Evaluation

**Fig. 5.**
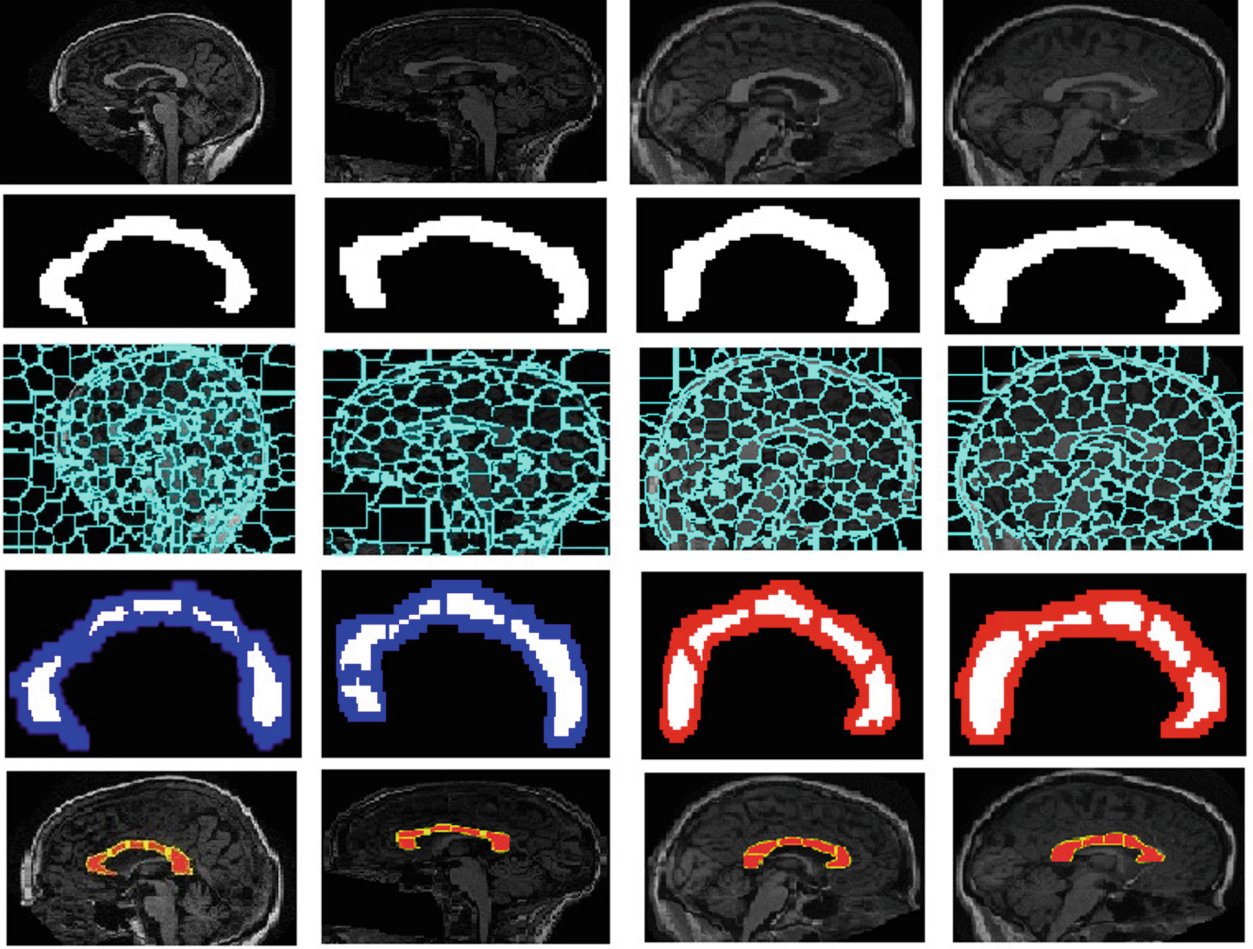
Experimental results: 1st line: Input image. 2nd line: Isolated CC. 3rd line: Brain parcellation. 4th line: Proposed CC parcellation. 5th line: Ground-truth.

For each subject, the proposed parcellation method gives an apparent variation in the positioning of the CC parcels. This is because this method is purely automatic and does not follow any atlas or any prior knowledge (Fig. 5). The geometric methods of Hofer and Witelson do not present the variation of their proportion of CC parcels and consequently, the same behavior can be observed on the results of all the subjects. Figure 5 shows that the proposed CC parcellation method is more similar to the Hofer parcellation than the Rittne one. This can be explained by the fact that Hofer subdivisions are based on the connections of the cortical fibers to find the CC parcels. The largest differences between the proposed parcellation and that of Witelson are observed in the parcels 1 and 4. In fact, according to our collaborator clinician expert, the CC shape and parcellation are well defined and the delineated CC area shows closely the five anatomical subdivisions of the CC, especially the critical ones: the rostrum and the splenium. The fornix is correctly removed from the CC area and the obtained CC parcellation shows a precise subdivision of CC into five regions within brain MRI scans, without penetrating the irrelevant neighboring structures. Note that, within the selected sample of MRI brain scans, the CC is extracted and parcelled both on female (column 1 and 3) and male (column 2 and 4) subjects. In fact, we applied the proposed method on subjects from the ABIDE dataset (column 1 and 2) as well as from the OASIS dataset (column 3 and 4).

### Quantitative Evaluation

In order to evaluate the performance of the proposed method, we used the following commonly used metrics: Dice, accuracy, sensitivity, specificity and precision.

- The Dice coefficient (5) is a statistical measure that is used for comparing the similarity of two sample sets.
- The accuracy (6) is defined as the rate of correctly classified items.
- The sensitivity (7) is the proportion of positive items correctly classified.
- The specificity (8) is the rate of negative items rightly identified.
- The precision (9) is the ratio of correctly predicted positive samples to the total predicted positive samples.

5 $$\begin{aligned} Dice = \frac{{2 \times TP}}{{2 \times TP + FN + FP}} \end{aligned}$$

6 $$\begin{aligned} Accuracy =\frac{(TP + TN)}{(TP + FN + TN + FP)} \end{aligned}$$

7 $$\begin{aligned} Sensitivity = \frac{TP}{(TP + FN)} \end{aligned}$$

8 $$\begin{aligned} Specificity = \frac{TN}{(TN + FP)} \end{aligned}$$

9 $$\begin{aligned} Precision = \frac{TP}{(TP + FP)} \end{aligned}$$

TP refers to the True Positive (region correctly parcelled as the concerned parcel), TN refers to the True Negative (region correctly classified as background), FP refers to the False Positive (region which is parcelled as the concerned parcel) and FN refers to the False Negative (region which is incorrectly classified as background). We notice that we produce five parcels, and for each parcel we measure the five metrics. It is worthy noting that for the first time, a very useful ground-truth for CC segmentation and parcellation within the challenging widely used OASIS and ABIDE datasets is used. Therefore, we are the only work that is compared to a such ground-truth. However, the Rittner method is evaluated only on the agreement between the results achieved by different CC parcellation methods. In fact, a professional neurologist from Pasteur Institute of Tunis and a junior doctor have been charged with manually preparing the CC regions and parcels from all images belonging to the OASIS and the ABIDE datasets. Besides, we applied post-processing in order to exclusively extract the CC area and parcels. Table 1 shows the recorded results comparatively to the ground-truth. It is clear that the Proposed Method (PM) records the higher Dice coefficient score ($>0.84$) in the parcels 1, 2, and 5, and a sufficient Dice coefficient score ($>0.75$) in parcels 3 and 4 comparatively to the ground-truth. Evenly, it reaches a higher accuracy, specificity and sensitivity scores with values $>0.90$. The decline of the proposed method performance according to the precision metric can be explained by the cause of the ground-truth which is manually drawing and the processing applied to do the evaluation in each parcel. Furthermore, for the two datasets and for each CC parcel, the Dice coefficient was computed pairwise for the methods of the state of the art (Table 2) as it is used in the Rittner work. Therefore, the previous analyzes allow only verifying the similarity between the resulting CC parcels, or which present statistical differences between methods of the literature since this is a problem without a gold standard (Table 2). Hence, it is now possible to know the correct CC parcellation by producing ground-truth for both Witelson and Hofer methods. Since the Hofer and Witelson CC parcellation methods are based on geometric CC parcellation, their results did not vary among different subjects throughout the experimented dataset. This explains this overlap measurement obtained which would have maximum value if any of the methods was the same. The most pertinent difference between these CC parcellation methods was related to the automatic and non-geometric behavior defined by our proposed parcellation. Table 2 presents different results between methods while recording interesting similarities in some cases. The proposed CC parcellation method demonstrates to be nearby to the Hofer method, mainly on parcels 1, 2 and 3, while the Witelson method presents significant statistical difference on the parcels 4 and 5.

**Table 1.** Evaluation of the proposed method.

	Dice	Accuracy	Sensitivity	Specificity	Precision
Parcel 1	0.9401	0.9986	0.9992	0.9986	0.7246
Parcel 2	0.8488	0.9927	0.9935	0.9927	0.4817
Parcel 3	0.7583	0.9944	0.9959	0.9944	0.5496
Parcel 4	0.7707	0.9960	0.9972	0.9960	0.6280
Parcel 5	0.8473	0.9889	0.9845	0.9890	0.3790
Mean$\pm std$	$0.8330\pm 0.050$	$0.9941\pm 0.003$	$0.9941\pm 0.001$	$0.9941\pm 0.013$	$0.5526\pm 0.363$

**Table 2.** Dice coefficient for the two datasets (best value are in bold).

	Witelson vs PM	Hofer vs. PM	PM vs. GT	Witelson vs. GT	Hofer vs. GT
Parcel 1	0.8512	0.9100	$\mathbf 0.9401$	0.6125	0.7013
Parcel 2	0.6001	0.7589	$\mathbf 0.8488$	0.2822	0.1624
Parcel 3	$\mathbf 0.8845$	0.8700	0.7583	0.4760	0.47163
Parcel 4	0.5113	0.5236	$\mathbf 0.7707$	0.4909	0.5120
Parcel 5	0.5112	0.4958	$\mathbf 0.8473$	0.6868	0.8014

## Conclusion

CC is the biggest fiber tract within the human brain that allows the communication between the two cerebral hemispheres. The CC form and sub-regions might cause some diseases. The CC parcellation from MRI images can predict future cases of diseases or progress neurological patterns in the development of different diseases. This paper presented a fully automatic non-geometric CC parcellation based on the SLIC superpixel algorithm, with no parameter adjusting and instantaneous training. Since there is no gold standard used to evaluate the existing methods, we produced for the first time a ground-truth led to evaluate quantitatively CC parcellation methods. Extensive experiments and quantitative comparisons with relevant CC parcellation methods, proved the accuracy of the proposed method on two challenging standard datasets. Indeed, the proposed method achieves higher performance values for each parcel. As future work, we aim to propose a super voxel method based on the SLIC algorithm, from not only MRI scans but also from functional magnetic resonance imaging.
